# Candida parapsilosis Oral Infection in an HIV-Negative Infant With Profound CD4+ Lymphopenia: Unveiling a Rare Immunodeficiency Scenario

**DOI:** 10.7759/cureus.87740

**Published:** 2025-07-11

**Authors:** Filippos Filippatos, Vasiliki Karava, Konstantinos Kakleas, Adina Santou, Athanasios Michos

**Affiliations:** 1 Pediatrics, First Department of Pediatrics, Medical School, National and Kapodistrian University of Athens, Aghia Sophia Children's Hospital, Athens, GRC; 2 Nephrology, Aghia Sophia Children's Hospital, Athens, GRC; 3 Allergy and Immunology, Aghia Sophia Children's Hospital, Athens, GRC; 4 Infectious Diseases, First Department of Pediatrics, Infectious Diseases and Chemotherapy Research Laboratory, Medical School, National and Kapodistrian University of Athens, Aghia Sophia Children's Hospital, Athens, GRC

**Keywords:** candida parapsilosis, cd4 lymphocytopenia, hiv testing, immunodeficiency, pediatrics

## Abstract

We describe a rare pediatric case of severe oral candidiasis caused by *Candida parapsilosis (C. parapsilosis),* associated with significant CD4^+^ lymphopenia and negative HIV serology. A previously healthy six-year-old female presented with high-grade fever and refractory oral lesions. Laboratory investigations revealed profound CD4^+^ lymphopenia and negative HIV serology. Treatment with intravenous fluconazole resulted in rapid clinical improvement. This report underscores the importance of early immunological evaluation and genetic investigation in pediatric patients with atypical oral candidiasis.

## Introduction

*Candida parapsilosis (C. parapsilosis)* has emerged as a significant fungal pathogen, predominantly affecting immunocompromised hosts, neonates, patients on prolonged antibiotic therapy, or individuals with compromised immune systems, including HIV-positive patients, transplant recipients, and those undergoing immunosuppressive treatments [1,2]. Oral candidiasis is a common mucosal fungal infection predominantly caused by frequently attributed to *Candida albicans. *Infections caused by non-albicans *Candida* species, such as *C. parapsilosis*, are particularly unusual in otherwise healthy pediatric populations and strongly suggest underlying immunodeficiency [3,4].

Primary immunodeficiency disorders (PIDs) are heterogeneous conditions characterized by impaired immune responses, predisposing patients to recurrent, severe, or atypical infections, autoimmunity, or malignancies. Profound CD4^+^ lymphopenia in HIV-negative children is exceedingly rare and often indicative of underlying conditions such as idiopathic CD4 lymphocytopenia (ICL), common variable immunodeficiency (CVID), or combined immunodeficiencies (CIDs). These disorders compromise mucosal immunity significantly, predisposing patients to severe fungal infections [5-7]. CD4^+^ T-cells are critical for effective immune responses against fungal pathogens, including mucosal defense against *Candida* species, thus rendering CD4-deficient individuals particularly susceptible to chronic or severe candidal infections [7-9]. Evidence in the literature highlights the need for early identification and characterization of immunodeficiencies to implement timely and effective interventions, reducing morbidity and improving patient outcomes [8,9].

The standard therapeutic approach for oral candidiasis typically involves topical antifungals, such as nystatin or clotrimazole, for mild cases. In moderate-to-severe or recurrent infections, systemic therapy with azoles, especially fluconazole, is preferred due to its favorable pharmacokinetics, tolerability, and effective mucosal penetration. However, increasing azole resistance among non-albicans *Candida* species, including *C. parapsilosis*, underscores the importance of performing antifungal susceptibility testing to tailor appropriate therapy and avoid clinical failure [10-12]. Alternative therapeutic options in cases of azole resistance or intolerance include echinocandins (e.g., caspofungin, micafungin) or amphotericin B formulations [10,12].

Given the rarity of severe *C. parapsilosis* mucosal infections in immunocompetent pediatric populations, identification of such cases should prompt clinicians to thoroughly evaluate for possible immunological defects and promptly initiate targeted antifungal therapy guided by susceptibility profiles. Early recognition and comprehensive management, including detailed immunological and genetic evaluations, can significantly improve prognosis, prevent complications, and guide long-term care strategies [3,5,9]. In this context, the unique clinical presentation of the patient in this case report highlights the critical importance of comprehensive immunological evaluations in pediatric patients with atypical oral infections.

## Case presentation

A previously healthy six-year-old female presented with a four-day history of persistent high-grade fever reaching up to 39.4 °C, severe oral pain, difficulty swallowing, significantly decreased oral intake, lethargy, irritability, mild weight loss, and decreased urine output. The patient had been initially assessed as an outpatient by a general pediatrician and diagnosed with pharyngotonsillitis and managed with amoxicillin and pain relief. However, a lack of treatment response and worsening of symptoms had prompted hospital admission for further evaluation and treatment.

**Figure 1:**
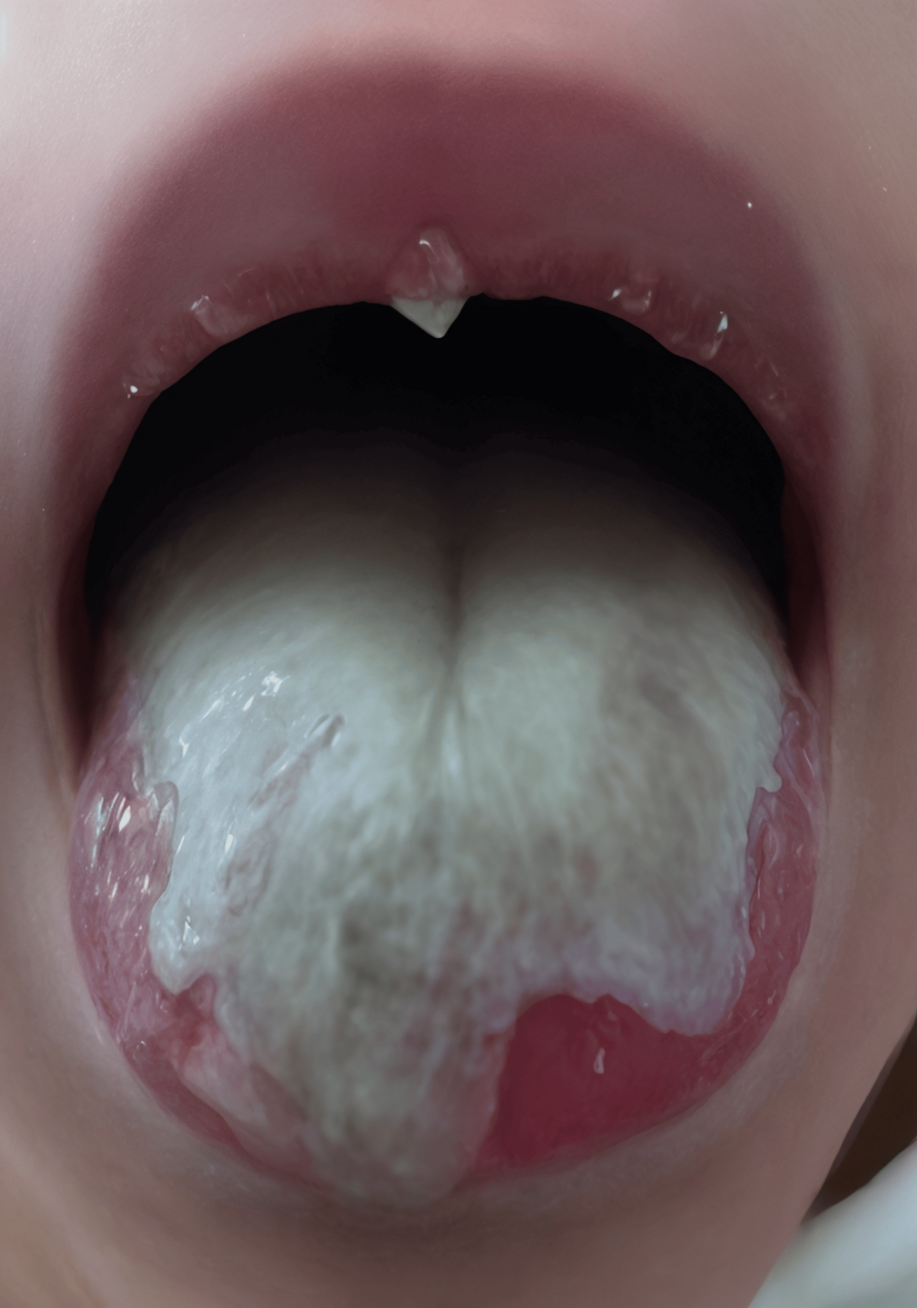
Oral candidiasis involving buccal mucosa and tongue The image shows an extensive, thick, white adherent plaques involving the buccal mucosa and dorsal tongue surface, characterized by significant surrounding mucosal erythema and edema, presence of superficial mucosal ulcerations, and minimal mucosal bleeding upon gentle manipulation, indicating severe inflammation and tissue fragility

Upon hospital admission, she appeared moderately ill, febrile (38.1 °C), mildly pale, tachycardic (heart rate: 122 bpm), with dry mucous membranes and decreased skin turgor indicative of mild dehydration. Detailed examination of the oral cavity showed severe candidiasis characterized by extensive, thick, adherent white plaques covering the tongue, buccal mucosa, and soft palate, with significant surrounding erythema, mucosal edema, tenderness, ulceration, and minimal mucosal bleeding on manipulation (Figure 1). Figure 1 shows extensive, thick, white adherent plaques involving the buccal mucosa and dorsal tongue surface, characterized by significant surrounding mucosal erythema and edema, presence of superficial mucosal ulcerations, and minimal mucosal bleeding upon gentle manipulation, indicating severe inflammation and tissue fragility.

Physical examination revealed no cervical or generalized lymphadenopathy, facial swelling, or rash. Capillary refill time was approximately two seconds, indicating adequate peripheral perfusion. Her nutritional status was mildly compromised, reflected by recent mild weight loss.

Initial laboratory evaluation demonstrated mild anemia (hemoglobin: 11.1 g/dL, normal range: 11.5-13.5 g/dL), leukocytosis (white cell count: 10.38 × 10³/µL, normal range: 4.5-13.5 × 10³/µL, neutrophils predominant) and elevated inflammatory markers [C-reactive protein: CRP: 47.4 mg/L, normal range: <5 mg/L]. Platelet count was within the normal range (295 × 10³/µL). Renal and liver function tests, as well as electrolyte levels, remained within normal limits (Table 1).

**Table 1:** Laboratory tests on admission ALT: alanine aminotransferase; AST: aspartate aminotransferase; CRP: C-reactive protein

Laboratory test	On admission	Reference range
Hemoglobin	11.1 g/dL	11.5–13.5 g/dL
Total leukocyte count	10.38 × 10³/µL	4.5–13.5 × 10³/µL
Platelet count	295 × 10³/µL	150–450 × 10³/µL
CRP	47.4 mg/L	<5 mg/L
CD4+ lymphocyte count	179 cells/µL	500–1500 cells/µL
Urea	28 mg/dL (normal)	10–40 mg/dL
Creatinine	0.4 mg/dL (normal)	0.3–0.7 mg/dL
AST	35 U/L (normal)	15–50 U/L
ALT	25 U/L (normal)	10–40 U/L
Sodium (Na)	138 mEq/L (normal)	135–145 mEq/L
Potassium (K)	4.2 mEq/L (normal)	3.5–5.0 mEq/L
Chloride (Cl)	102 mEq/L (normal)	98–107 mEq/L

Due to the atypical clinical presentation of severe oral stomatitis, an extensive immunological workup was performed. Peripheral blood immunophenotype test by flow cytometry was performed and showed a significant reduction in CD4+ lymphocyte count (179 cells/µL, normal range: 500-1500 cells/µL). Other lymphocyte subsets, including CD8+ T cells (850 cells/µL, reference range: 300-1000 cells/µL), B cells (520 cells/µL, reference range: 200-600 cells/µL), and natural killer (NK) cells (250 cells/µL, reference range: 90-590 cells/µL), were within normal limits. Immunoglobulin levels were also normal: IgG: 1020 mg/dL (reference range: 700-1600 mg/dL), IgA: 95 mg/dL (reference range: 70-400 mg/dL), and IgM: 85 mg/dL (reference range: 50-250 mg/dL). Complement factors C3 (120 mg/dL, reference range: 80-160 mg/dL) and C4 (25 mg/dL, reference range: 15-45 mg/dL) were normal. Vaccine response assessments showed protective antibody titers against tetanus (>1.0 IU/mL; protective: ≥0.1 IU/mL), diphtheria (>1.0 IU/mL; protective: ≥0.1 IU/mL), hemophilus influenzae type b (anti-Hib antibodies: 2.5 µg/mL; protective: ≥1.0 µg/mL), hepatitis B surface antibody (120 IU/L; protective: ≥10 IU/L), measles (positive), and pneumococcal conjugate vaccine serotypes (adequate protective levels for age). HIV serology remained negative.

Microbiological evaluations, which included multiple blood cultures, throat swabs for bacterial and viral pathogens [influenza, adenovirus, Epstein-Barr virus (EBV), Cytomegalovirus (CMV)], and specific serology for HIV, hepatitis B and C, syphilis, and toxoplasmosis, were negative (Table 2).

**Table 2:** Microbiological evaluation workup CMV: cytomegalovirus; EBV: Epstein-Barr virus; HIV: human immunodeficiency virus

Microbiological test	Specimen type	Result
Blood cultures	Blood	Negative
Bacterial pathogens	Throat swab	Negative
Influenza	Throat swab	Negative
Adenovirus	Throat swab	Negative
EBV	Throat swab	Negative
CMV	Throat swab	Negative
HIV serology	Blood	Negative
Hepatitis B serology	Blood	Negative
Hepatitis C serology	Blood	Negative
Syphilis serology	Blood	Negative
Toxoplasmosis serology	Blood	Negative
Fungal culture	Oral lesions	*Candida parapsilosis* (borderline fluconazole resistance)

Oral lesion cultures identified *C. parapsilosis*, exhibiting borderline resistance to fluconazole (Table 3).

**Table 3:** Antifungal susceptibility test results MIC: minimum inhibitory concentration

Antifungal agent	MIC (μg/mL)	Interpretation
Amphotericin B	0.5	Susceptible
Fluconazole	1	Susceptible (dose-dependent/borderline)
Itraconazole	0.03	Susceptible
Isavuconazole	-	Not reported
Voriconazole	0.008	Susceptible
Posaconazole	0.015	Susceptible
Caspofungin	0.125	Susceptible
Anidulafungin	0.25	Susceptible
Micafungin	0.125	Susceptible
Flucytosine	0.06	Susceptible

Following infectious disease consultation and given the normal renal and liver function tests (creatinine: 0.4 mg/dL, AST: 35 U/L, ALT: 25 U/L), intravenous fluconazole (6 mg/kg/day) was initiated alongside supportive care, including analgesics for pain management (paracetamol), and nutritional support with intravenous fluids and hypercaloric oral supplements.

Clinical improvement was prompt; fever resolved within 48 hours, oral lesions and mucosal inflammation significantly improved by day four, and oral intake gradually restored within one week. The comparison of laboratory values on admission and after therapeutic intervention is presented in Table 4.

**Table 4:** Laboratory tests on admission and after therapeutic intervention ALT: alanine aminotransferase; AST: aspartate aminotransferase; CRP: C-reactive protein

Laboratory test	Reference range	On admission	After intervention
Hemoglobin	11.5–13.5 g/dL	11.1 g/dL	12.3 g/dL (normal)
Total leukocyte count	4.5–13.5 × 10³/µL	10.38 × 10³/µL	8.2 × 10³/µL (normal)
Platelet count	150–450 × 10³/µL	295 × 10³/µL	310 × 10³/µL (normal)
CRP	<5 mg/L	47.4 mg/L	2.3 mg/L (normal)
CD4+ lymphocyte count	500–1500 cells/µL	179 cells/µL	620 cells/µL (normal)
Urea	10–40 mg/dL	28 mg/dL (normal)	25 mg/dL (normal)
Creatinine	0.3–0.7 mg/dL	0.4 mg/dL (normal)	0.5 mg/dL (normal)
AST	15–50 U/L	35 U/L (normal)	30 U/L (normal)
ALT	10–40 U/L	25 U/L (normal)	22 U/L (normal)
Sodium (Na)	135–145 mEq/L	138 mEq/L (normal)	140 mEq/L (normal)
Potassium (K)	3.5–5.0 mEq/L	4.2 mEq/L (normal)	4.1 mEq/L (normal)
Chloride (Cl)	98–107 mEq/L	102 mEq/L (normal)	104 mEq/L (normal)

Given the severity of the initial presentation, the borderline susceptibility (MIC of 1 µg/mL) of *C. paraprsilosis*, and the documented underlying CD4+ lymphopenia (indicating immune compromise), it was deemed optimal to continue oral fluconazole (6 mg/kg/day) therapy for two additional weeks post-discharge in line with current clinical guidelines for the management of mucosal candidiasis in immunocompromised pediatric patients [10].

The patient was diagnosed clinically with profound isolated CD4+ lymphopenia of suspected primary immunodeficiency origin [likely ICL or another genetically driven CID], pending definitive genetic confirmation. Scheduled comprehensive genetic and immunological evaluations included immunoglobulin levels, vaccine response assessments, genetic counseling, and follow-up immunological consultations to monitor immune recovery and identify underlying genetic or immune deficiencies.

## Discussion

*C. parapsilosis* commonly causes invasive infections in neonates, critically ill, or immunocompromised children, particularly those with central venous catheters or extended hospital stays [1-3]. Isolation of *C. parapsilosis* from oral lesions in a previously healthy child, as reported here, is exceptionally unusual and highlights potential undiagnosed immunological dysfunction [2,5]. Previous pediatric cases involving non-albicans *Candida* infections, particularly *C. parapsilosis*, generally report underlying risk factors such as immunosuppressive therapy or extensive hospitalizations, unlike this patient's presentation [2,3]. Comparing these cases emphasizes the uniqueness of this patient's severe candidiasis in the absence of common risk factors, suggesting a novel immunological defect.

Azole resistance in *C. parapsilosis *is increasingly recognized and linked to mechanisms such as mutations in the ERG11 gene, overexpression of efflux pumps, and biofilm formation, complicating therapeutic management. The borderline resistance seen in this case reinforces the importance of routine antifungal susceptibility testing to guide treatment effectively [7-10]. Profound CD4+ lymphopenia without HIV infection in pediatric patients is exceedingly rare and often indicates significant underlying immunological abnormalities [5-7]. CD4+ T-cells play a critical role in mucosal immunity, which is essential for effective responses against fungal pathogens, including *Candida* species. Their depletion increases vulnerability to invasive and persistent infections, as observed in this case [3-6]. Long-term implications of chronic CD4+ lymphopenia include heightened susceptibility to recurrent infections, autoimmune conditions, and potentially increased malignancy risk, necessitating careful and prolonged follow-up. Preventive strategies, including prophylactic antimicrobial therapy, routine vaccination updates, and regular monitoring for autoimmune and malignant diseases, are recommended [3-5].

The limited literature on pediatric cases combined with insights from adult studies provides valuable guidance for clinicians encountering atypical presentations of *C. parapsilosis* infections in HIV-negative children, prompting careful immunological and microbiological evaluation. The existing pediatric literature primarily reports invasive infections associated with significant predisposing factors, such as prolonged hospitalization, immunosuppressive therapy, or surgical interventions [3,13,14,15]. Case reports describing severe mucosal or systemic infections with *C. parapsilosis* in previously healthy children without overt risk factors or HIV infection remain exceedingly rare, highlighting unique underlying immune dysfunction scenarios similar to the case presented here [16,17]. A comparative summary table contrasting key clinical features, underlying conditions, and outcomes from similar pediatric case reports available in the literature is presented in Table 5.

**Table 5:** Comparative summary table contrasting key clinical features, underlying conditions, and outcomes from similar pediatric case reports in the literature HIV: human immunodeficiency virus; ICU: intensive care unit

Study	Age/sex	Clinical features	Underlying conditions/immunodeficiency	Treatment	Outcome
Mantadakis and Tragiannidis, 2019 [3]	2 years/female	Severe oral and esophageal candidiasis	Prolonged ICU stay, corticosteroid therapy	Fluconazole, caspofungin	Recovery after prolonged antifungal therapy
Dotis et al., 2012 [15]	4 years/male	Fever, bloodstream infection (fungemia)	Immunosuppressive therapy (chemotherapy for leukemia)	Amphotericin B, caspofungin	Complete recovery, resolution of candidemia
Mohan et al., 2015 [16]	5 years/male	Persistent fever, oral thrush, poor intake	Previously healthy, transient lymphopenia of unclear etiology	Amphotericin B, fluconazole	Complete recovery, normal immune function
Inacio et al., 2017 [17]	8 months/female	Extensive mucocutaneous candidiasis, failure to thrive	Previously healthy, no definitive immunodeficiency identified	Fluconazole, topical antifungals	Improvement with prolonged antifungal therapy
Current case (2025)	6 years/female	Severe oral candidiasis, high-grade fever, poor intake	Isolated profound CD4+ lymphopenia (179 cells/µL), HIV-negative, suspected primary immunodeficiency	IV and oral fluconazole	Clinical improvement, awaiting genetic evaluation

Data in adult populations suggest that infections with non-albicans *Candida* species, including *C. parapsilosis*, often occur in the context of reduced cellular immunity, prolonged hospital stays, broad-spectrum antibiotic exposure, or prior antifungal treatment [1,9]. Furthermore, increasing recognition of fluconazole resistance mechanisms among *C. parapsilosis* isolates in adults, including ERG11 gene mutations and efflux-pump overexpression, emphasizes the importance of antifungal susceptibility testing to guide pediatric management [11,17]. Such evidence underscores the necessity for comprehensive immunological evaluation and susceptibility-guided antifungal therapy in pediatric cases to optimize therapeutic outcomes.

Beyond HIV-driven immunodeficiency, several alternative factors could have contributed to the profound CD4+ lymphopenia and severe oral candidiasis observed in this patient. Genetic predispositions, such as ICL, CVID, or CIDs, may underlie isolated CD4+ deficiencies, particularly when standard infectious etiologies have been excluded. While extensive microbiological screening in this patient excluded active coinfections, previous infections or transient viral suppressions of cellular immunity could also theoretically contribute. Furthermore, although no recent immunosuppressive medications were documented in the patient's history, clinicians should always consider medication-induced lymphopenia. Lastly, mild nutritional compromise and dehydration observed at admission may have further influenced the immune response, highlighting the multifactorial nature of such clinical presentations.

Comprehensive genetic and immunological screening in similar pediatric cases should involve assessments of immunoglobulin levels, specific vaccine responses, lymphocyte subset analysis, and genetic tests targeting primary immunodeficiencies such as ICL, CVID, and CID syndromes. Multidisciplinary collaboration involving immunologists, geneticists, infectious disease specialists, and pediatricians is essential for optimal patient management and outcomes [11-13].

## Conclusions

This report underscores the diagnostic challenge posed by severe oral candidiasis caused by *C. parapsilosis *in an HIV-negative infant without apparent risk factors. Clinicians should maintain a high index of suspicion for underlying primary immunodeficiencies when encountering atypical fungal infections. Early recognition, prompt antifungal therapy guided by susceptibility testing, and timely referral for genetic and immunological evaluation are essential steps to prevent severe complications and improve patient outcomes.
